# Early Platelet Dysfunction in Sepsis: An ICU Pilot Study

**DOI:** 10.3390/pathogens15020196

**Published:** 2026-02-10

**Authors:** Maria Grazia Bocci, Silvia Sorrentino, Ilaria Gatto, Daniele Natalini, Emiliano Cingolani, Allegra Blandina, Francesca Botta, Manfred Caravella, Simone Carelli, Domenico Luca Grieco, Alessandra Ionescu Maddalena, Luca D’Innocenzo, Matteo De Siati, Riccardo Maviglia, Chiara Gori, Erica De Candia

**Affiliations:** 1 Department of Clinical and Clinical Research, National Institute for Infectious Diseases Lazzaro Spallanzani, IRCCS, Via Portuense 292, 00149 Rome, Italy; mariagrazia.bocci@inmi.it; 2 Unit of Haemostasis and Thrombosis Diseases, Fondazione ‘Policlinico Universitario A. Gemelli’ IRCCS, L.go A. Gemelli 8, 00168 Rome, Italy; silvia.sorrentino@policlinicogemelli.it (S.S.); ilariagatto@yahoo.it (I.G.); luca.d.innocenzo95@gmail.com (L.D.); m.desiati97@gmail.com (M.D.S.); erica.decandia@unicatt.it (E.D.C.); 3 Department of Emergency, Intensive Care Medicine and Anesthesia, Fondazione Policlinico Universitario A. Gemelli IRCCS, 00168 Rome, Italy; daniele.natalini@policlinicogemelli.it (D.N.); allegra.blandina09@gmail.com (A.B.); francesca.botta@yahoo.it (F.B.); manfredcaravella@outlook.it (M.C.); simone.carelli@policlinicogemelli.it (S.C.); domenicoluca.grieco@unicatt.it (D.L.G.); riccardo.maviglia@policlinicogemelli.it (R.M.); 4 Department of Anesthesiology and Intensive Care Medicine, Catholic University of the Sacred Heart, Fondazione ‘Policlinico Universitario A. Gemelli’ IRCCS, L.go F. Vito, 00168, Rome, Italy; 5 UOC Shock & Trauma, Azienda Ospedaliera San Camillo Forlanini, C.ne Gianicolense 87, 00152 Rome, Italy; ecingolani@scamilloforlanini.rm.it; 6 UOC Anesthesia and Intensive Care Medicine, Polo Ospedaliero Belcolle, 01100 Viterbo, Italy; alessandra.ionescumaddalena@asl.vt.it; 7 Department of Translational Medicine and Surgery, Catholic University of the Sacred Heart, 00168 Rome, Italy

**Keywords:** sepsis, platelet dysfunction, thrombocytopenia, TEG, procalcitonin, SOFA score

## Abstract

Platelets are critical for hemostasis and play an active role in immune responses to infection. While thrombocytopenia in sepsis is associated with poor outcomes, platelet dysfunction remains less explored. This prospective observational pilot study investigated the relationship between platelet dysfunction and sepsis severity using multiple platelet function tests. Ten adults with sepsis or septic shock admitted to the ICU of “Fondazione Policlinico Universitario A. Gemelli” and seven healthy controls were enrolled. Blood samples were collected at admission (T0), after 48 h (T1), and after 7 days (T2). Controls were sampled only at T0. Besides platelet count, hemostatic platelet function was assessed by light transmission aggregometry (LTA), thromboelastography (TEG), and platelet activation markers (P-selectin and PAC-1 expression), whereas immune platelet function was assessed by investigation of platelet–leukocyte aggregates and soluble plasma levels of CD40L. Platelet function was correlated with procalcitonin levels and SOFA scores. While thrombocytopenia developed after 48 h, hemostatic and immune platelet dysfunctions were already evident at T0. Platelet function abnormalities were correlated with sepsis severity, as reflected by higher SOFA scores and elevated procalcitonin levels, particularly at T0. Early platelet dysfunction, preceding thrombocytopenia, may represent a potential early indicator of sepsis severity and support timely intervention for hemostatic and immune platelet-dependent abnormalities in septic patients.

## 1. Introduction

Sepsis and septic shock represent significant global health challenges with high mortality rates, particularly in intensive care units, where over 40% of affected patients die. Early diagnosis and appropriate clinical management are crucial in improving survival rates, much like the treatment of severe trauma or myocardial infarction [1,2]. Coagulopathy plays a crucial role in the progression of sepsis to septic shock and multi-organ failure (MOF) [3] and is considered an independent risk factor for worse outcomes [4]. Thrombocytopenia is the most prevalent hemostasis disorder, affecting 30–40% of patients and significantly increasing the risk of bleeding, especially when platelet counts fall below 100 × 10^9^/L [5,6]. It is considered an early sign of sepsis and is typically related to disease severity and poor prognosis [7,8,9]. In sepsis patients, thrombocytopenia may result from several factors, including platelet destruction, blood dilution, increased spleen activity, or decreased bone marrow production [6]. Moreover, the inflammatory response may enhance platelet adhesion to endothelial cells, contributing to a reduction in circulating platelet levels [10].

In addition to a reduced platelet count, septic patients also exhibit platelet function abnormalities, which are likewise associated with disease severity [11,12]. Platelets can exhibit hemostatic dysfunction that can manifest in two patterns: hyper-reactive and hypo-reactive. However, no current diagnostic tests are able to discriminate between the two states. In addition, the factors that determine the development of one pattern over the other remain unclear.

Over the past two decades, it has become well established that platelets possess multiple functions beyond their classical role in hemostasis and thrombosis, acting as key mediators of immune modulation [13,14] and inflammatory processes [15,16] through activation of white blood cells and interaction with endothelial cells [17]. These multifactorial properties of platelets become particularly relevant in the context of infections and sepsis. In fact, platelets release immune and proinflammatory mediators such as soluble CD40L, interact with endothelial cells to promote vascular permeability and inflammatory signaling, and form aggregates with neutrophils and monocytes, thereby facilitating neutrophil extracellular trap (NET) formation for pathogen trapping. They also support microvascular clot formation to confine pathogens, contributing to the process known as *“immunothrombosis”* [18,19]. During infection, this mechanism is beneficial: acute-phase proteins and procoagulant effects help eliminate pathogens by trapping them within localized clots formed through platelet interactions [17]. However, in sepsis, this localized immunothrombotic response becomes systemic and dysregulated, leading to disseminated intravascular coagulation (DIC) and multi-organ dysfunction syndrome (MODS) [20]. Among several other effectors, two platelet molecules—P-selectin and CD40L—play a crucial role in activating both innate and adaptive immunity. These molecules are stored in α-granules and, upon platelet activation, are expressed on the platelet membrane and released in plasma. Both serve as key mediators of platelet interactions with leukocytes, thereby facilitating immune cell recruitment and signaling.

P-selectin interacts with the P-selectin receptor (PSGL-1) expressed on leukocytes, particularly neutrophils [21]. Instead, CD40L is a soluble inflammatory mediator that binds the CD40 receptor on the surface of numerous cells, including immune cells, leading to leukocyte activation and leukocyte-platelet aggregates. It also appears to play a role in the maturation of B cells and dendritic cells [22,23,24].

Although platelets are involved in sepsis due to both their hemostatic and immune functions, their functional assessment is not routinely performed due to the complexity of platelet function testing and the limited availability of such assays. Hemostasis is still commonly assessed using standard laboratory tests such as prothrombin time (PT), activated partial thromboplastin time (aPTT), fibrinogen, D-dimer, and platelet count. As widely recognized, alterations in these parameters are closely associated with the severity of sepsis [1]. However, these tests only provide a partial investigation of the haemostatic process, as they focus exclusively on coagulation pathways, ruling out the role of cellular components such as platelets and endothelial cells. From this perspective, bedside viscoelastic tests, such as thromboelastography (TEG), which analyzes the entire coagulation process, offer a more comprehensive assessment of coagulation status and a more sensitive detection of platelet disease in 20–30 min [25]. Studies have shown that integrating standard coagulation parameters with viscoelastic improves the assessment of prognosis and disease severity [26]. Thromboelastography (TEG) assesses the clot formation process from initiation to fibrinolysis [27,28], providing parameters that reflect the individual contributions of coagulation factors, platelets, and fibrin at various stages of the coagulation cascade [29]. Among these parameters, the maximum amplitude (MA) provides a comprehensive evaluation of both the quantity and functional status of fibrinogen and platelets involved in clot formation, making it a reliable indicator of platelet function [30]. Platelet Mapping, a specific type of TEG analysis, is used to test platelet function in clot generation exclusively using platelet activators such as ADP and arachidonic acid (AA). MA, the primary parameter analyzed in this study, is a measurement of clot stiffness with the contribution of fibrin and platelets. This parameter is commonly used to detect the presence of antiplatelet agents that inhibit platelet function. For example, P2Y12 inhibitors lead to a reduction in ADP-induced MA (MA_ADP_), while aspirin results in a decreased arachidonic acid-induced MA (MA_AA_).

The aim of our study is to evaluate hemostatic platelet function in patients with sepsis and septic shock using different laboratory methods and to assess the correlation between platelet function and sepsis severity, as measured by procalcitonin levels and SOFA score. Additionally, we investigated some pathways of expression of platelet immune response to pathogens, such as soluble CD40L and platelet–leukocyte aggregates, and markers of endothelial damage, such as circulating levels of von Willebrand factor.

We hypothesized that platelet dysfunction may occur in patients with sepsis or septic shock even when platelet counts are normal and that it could be detected early at disease onset. Furthermore, we hypothesized that platelet dysfunction might correlate with sepsis severity indicators, thus highlighting the role of platelet function—beyond platelet count alone—in the complex pathophysiology of sepsis and septic shock, as well as its potential value as a prognostic marker.

## 2. Materials and Methods

### 2.1. Study Design

This prospective observational study enrolled patients with sepsis and septic shock admitted to the intensive care unit of “Fondazione Policlinico Universitario A. Gemelli”, Rome, Italy. Sepsis and septic shock, as defined by the latest Sepsis-3 criteria, were managed according to the more recent recommendations of the Surviving Sepsis Campaign guidelines. Inclusion criteria were as follows: age over 18 years old, diagnosis of sepsis or septic shock from Gram-negative and Gram-positive bacteria supported by microbiological isolations and/or increased sepsis markers (procalcitonin and C-reactive protein [CRP]), and an expected length of stay of more than 48 h. Exclusion criteria were as follows: history of bleeding and hemostasis disorder, uncontrolled diabetes, severe malnutrition (body weight on admission ≤ 45 kg, BMI ≤ 18.5), an expected length of stay in ICU less than 48 h, antiplatelet therapy administration in the previous 7 days, liver cirrhosis, and chronic inflammatory diseases. A control group was composed of healthy volunteers. The study was approved by the institutional ethics committee of Fondazione Policlinico Universitario A. Gemelli. Informed consent was obtained from the patients or, if they could not provide it, from their legal guardians.

### 2.2. Blood Collection

Blood samples were obtained from the patients at the time of admission (T0) in the intensive care unit, after 48 h (T1), and after 7 days (T2). The rationale for selecting these time points for sampling was based on the temporal dynamics of sepsis and septic shock progression and platelet behavior during the systemic inflammatory response, as supported by existing literature [31,32]. A single blood sample was taken from control subjects at the time of a new patient enrollment. Whole blood (WB) was collected through sampling from a venous or an arterial catheter into EDTA vacutainer tubes for CBC; into 3.8% sodium citrate vacutainer tubes for standard coagulation tests, von Willebrand tests, flow cytometry, LTA, thromboelastography, and platelet mapping assays; and into a vacutainer with no anticoagulant for soluble P-selectin and CD40L assays. All samples were processed within two hours from blood draw to ensure platelet integrity and to minimize ex vivo activation.

### 2.3. Blood Count

Platelet count and mean platelet volume (MPV), white blood cells, red blood cells, and hemoglobin levels were obtained from EDTA-anticoagulated samples using a Sie-mens ADVIA 2120 instrument (Siemens, Erlangen, Germany).

### 2.4. Light Transmission Aggregometry (LTA)

Platelet aggregation was measured using platelet-rich plasma (PRP) and LTA. Whole blood specimens were centrifuged at 140× *g* for 15 min to obtain PRP and at 2700× *g* for 15 min to obtain platelet-poor plasma (PPP). PRP was stirred at 600 rpm at 37 °C in an optical aggregometer (AggRAM Helena Bioscience Europe, Gateshead, UK), and aggregation was induced by adding 2 µM ADP (Helena Bioscience Europe, Gateshead, UK) or 5 µM (Helena Bioscience Europe, Gateshead, UK) epinephrine to the final concentrations. Aggregation was measured as a percentage change in light transmission. Only samples with a platelet count > 150 × 10^9^/L in PRP were analyzed.

### 2.5. Platelet Mapping Assay

The Platelet MappingTM assay was carried out on fresh (WB) samples following the instructions provided by the manufacturer using TEG6s (Haemonetics Corporation, Boston, MA, USA). Using Platelet Mapping, platelet activation was assessed through the cyclooxygenase 1 pathway by measuring the maximum amplitude (MA) of the clot in response to arachidonic acid (AA), as well as through the P2Y12 receptor pathway by measuring the MA in response to adenosine diphosphate (ADP). The results are ex-pressed as MAAA and MAADP and then as the percentage of platelet aggregation in re-sponse to arachidonic acid (%AA PM) and to ADP (%ADP PM).

### 2.6. Measurement of Platelet Activation Markers P-Selectin and Pac-1

Platelet activation and secretion markers were investigated by flow cytometry on WB samples before and after stimulation of platelets with specific agonists. WB samples were incubated with anti-CD42b-PECyc5 MoAb (BD Bioscience, Franklin Lakes, NJ, USA) to identify platelet population, mouse anti-human CD62P-PE (BD Biosciences, San Jose, CA, USA) to identify P-selectin, and mouse PAC-1-FITC (BD Biosciences, San Jose, CA, USA) to identify the fibrinogen receptor in its activated form. The binding of the above MoAbs was measured on qui-escent platelets and on platelets stimulated with 10 µM ADP and 25 µM thrombin re-ceptor-activating peptide (TRAP). Samples were analyzed by flow cytometer FC 500 (Beckman Coulter, Brea, CA, USA) and expressed as a percentage of P-selectin or PAC-1 positive platelets. Data were analyzed by CXP software (CytExpert for DxFLEX.Ink, Beckman Coulter, Brea, CA, USA).

### 2.7. Measurement of Circulating Platelet/Leukocyte Aggregates

Leukocyte-platelet aggregates were investigated by flow cytometry using the following MoAbs: anti-CD42b-PECyc5 to identify platelet population, anti-CD45 FITC MoAbs (Beckman Coulter), anti-CD-16 FITC (Beckman Coulter), and anti-CD 14 FITC (Beckman Coulter) to identify the percentage of leukocyte (CD45+)/platelet aggregates, neutrophil (CD 16+)/platelet aggregates, and monocyte (CD 14+)/platelet aggregates. Samples were analyzed by flow cytometer FC 500 and expressed as a percentage of CD45, CD16, or CD14 positive platelets. Data were analyzed by CXP software.

### 2.8. Soluble P-Selectin and CD40 Ligand Measurement

Samples drawn in tubes without anticoagulants were centrifuged at 2000× *g* for 10 min at 4 °C. The serum was then stored in aliquots at −80 °C until analysis.

Measurement of soluble P-selectin and soluble CD40 ligand in serum was per-formed using the anti-human P-selectin (R&D System, Inc., Minneapolis, MN, USA) and human CD40L/TNFSF5 (R&D System, Inc., Minneapolis, MN, USA) immunoassay kits ac-cording to the manufacturer’s instructions.

### 2.9. Measurement of Plasma Von Willebrand Factor (Vwf) Antigen and of Ristocetin Cofactor

Measurement of von Willebrand factor antigen and ristocetin cofactor was per-formed on plasma samples using an immuno-turbidimetric method (HemosIL AcuStar vWF Antigen e RiCof) on an AcuStar ACL system (Werfen, Milan, Italy), following the manufacturer’s in-structions.

### 2.10. Statistical Analysis

Continuous variables were expressed as median and interquartile range. Categorical variables were expressed as percentages. The Mann–Whitney test was used to statistically analyze differences between patients and controls, and Bonferroni correction was applied. The Friedman test was used to study the temporal trend of the patients’ platelet counts across the three time points. Correlation between variables was studied with the Spearman correlation coefficient. *p* < 0.05 was considered statistically significant. Statistical analysis was performed with the SPSS 26.0 System (IBM SPSS Statistics for Windows, Version 26.0, Armonk, NY, USA).

The sample size for this study was not formally calculated prior to data collection because the experimental procedures involved were highly time-consuming and resource-intensive. Enrollment in the study ruled out patients under antiplatelet treatment at the time of admission, thus further limiting the number of patients to be included. As a result, the study was designed with a feasible number of samples that balanced scientific rigor with logistical limitations. This approach, while limiting statistical power to some extent, allowed for meaningful preliminary insights. Furthermore, the recruitment of healthy control participants was based on voluntary enrollment, which constrained the sample size for this group.

Due to the limited sample size and the primary aim of our study (namely, the early assessment of platelet function), we decided to include both patients with sepsis and those with septic shock in the same study cohort, acknowledging that septic shock typically represents a more advanced stage of the disease. Given the early onset of platelet dysfunction in sepsis, we also considered it appropriate to include patients exhibiting signs of sepsis.

## 3. Results

### 3.1. Clinical and Laboratory Data of Patients

A total of ten patients (Table 1) and seven healthy volunteers were enrolled in the study. Six patients met the criteria for septic shock, while the remaining four patients were classified as having sepsis without shock.

Gram-negative bacteria were the predominant cause of infection in 70% of patients, whereas Gram-positive bacteria accounted for the remaining 30%. In 70% of the cases, the bacteria responsible for the infection were isolated from the blood, 10% from the lung, and 20% from other biological material (biopsy or liquid samples taken dur-ing source control surgery). The control group included four males and three females with a median age of 31 years (IQ 30.5–33.5). Clinical and laboratory data of enrolled patients are shown in Table 1.

### 3.2. Platelet Counts

The median platelet count in patients with sepsis and septic shock was not significantly different at T0 from that of healthy volunteers (227,000/µL [99.000–459.000] and 188.000/µL [156.500–214.000], respectively, *p* = 0.06).

**Figure 1:**
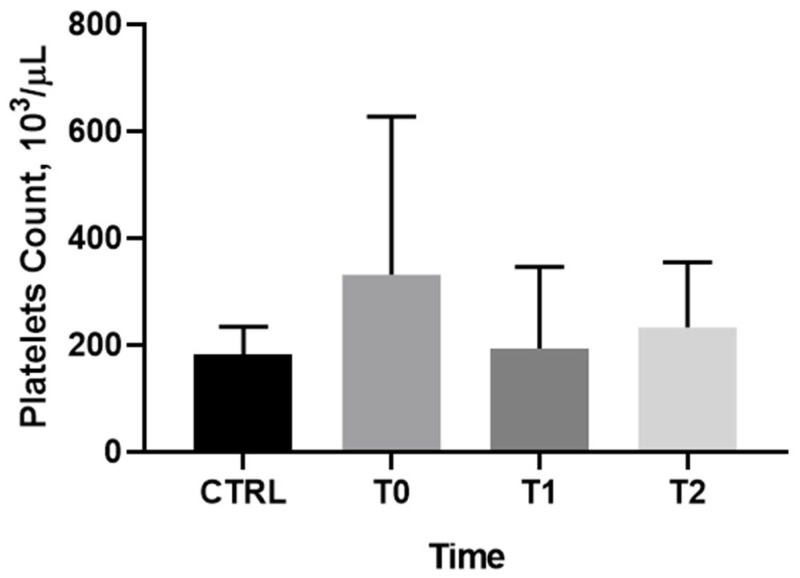
Platelet count of controls at T0 and patients’ platelet count at T0, T1, and T2.

Hence, thrombocytopenia did not occur early at the onset of sepsis or septic shock. Platelet count progressively decreased during the following days, with the most significant reduction occurring at 48 h (Figure 1).

Statistical analysis of patients’ platelet counts across the three time points (T0, T1, and T2) did not reveal a statistically significant difference over time (*p* > 0.05) nor versus controls.

### 3.3. Light Transmission Aggregometry

**Figure 2:**
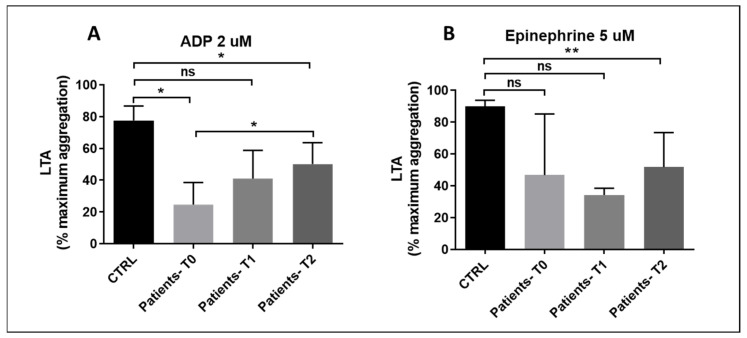
(**A**,**B**): (**A**) Patient platelet aggregation measured by light transmission aggregometry (LTA) after stimulation with ADP 2 µM at times T0, T1, and T2. T0 vs. CTRL: p*_bonf_* = 0.0380; T1 vs. CTRL: p*_bonf_* = 0.0952; T2 vs. CTRL: p*_bonf_* = 0.0328; T0 vs. T2: p*_bonf_* = 0.0484. (**B**) Platelet aggregation measured by light transmission aggregometry (LTA) after stimulation with EPI 5 µM at times T0, T1, and T2. T0 n = 4, T1 n = 3, T2 n = 7. T0 vs. CTRL: p*_bonf_* = 0.0909; T1 vs. CTRL: p*_bonf_* = 0.0714; T2 vs. CTRL: p*_bonf_* = 0.0066. Bonferroni correction was applied for all pairwise comparisons; *p*-values (p*_bonf_*) are reported. Sample size: n = 4, at T0, n = 3 at T1, n = 7 at T2. * *p* < 0.05, ** *p* < 0.01. ns = no significance.

LTA was performed in sepsis and septic shock patients at different times only when their platelet count in PRP was greater than 150,000/µL. For this reason, 6%, 7%, and 3% of all samples at T0, T1, and T2, respectively, were excluded from the analysis. Controls were investigated only once at the time of enrollment of each patient, e.g., T = 0. A significant reduction in platelet aggregation in response to 2 µM ADP was observed in patients with sepsis or septic shock at T0 and T2 compared with the control group, despite the small sample size. (Figure 2A,B). In addition, there was a significant improvement of ADP-induced LTA at day 7 (T0 vs. T2). Aggregation induced by 5 µM epinephrine showed a significant reduction at T2. A trend toward reduced aggregation was observed at T0 and T1, though these differences were not statistically significant, likely due to the small sample size. These results indicate that a defect in platelet function was present early in the disease onset and persisted for at least 7 days.

### 3.4. Viscoelastic Tests

**Figure 3:**
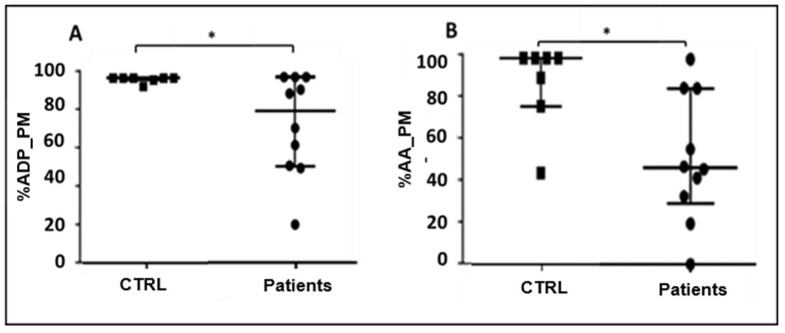
(**A**,**B**) Comparison of median and interquartile range values of ADP-induced (%ADP_PM) (* *p* = 0.027) and arachidonic acid-induced (% AA_PM) (* *p* = 0.030) platelet aggregation rates between controls and patients at T0.

We measured two parameters using the viscoelastic test Platelet Mapping: the platelet aggregation rate and the maximal amplitude (MA) in response to ADP and arachidonic acid. The percentage of platelet aggregation reflects the capacity of platelets to respond to specific agonists and to form aggregates in the early phases of platelet activation, whereas the maximal amplitude (MA) parameter is a parameter that indicates the overall clot strength depending not only on platelet aggregation but also on platelet–fibrin interaction, fibrinogen levels, and fibrin polymerization with cross-linking activity. Platelet function, investigated by measuring the percentage of platelet aggregation by both ADP (%ADP_PM) and arachidonic acid (%AA_PM), showed a significant impairment of platelet function in sepsis and septic shock patients compared to controls at T = 0 (Figure 3A,B).

**Figure 4:**
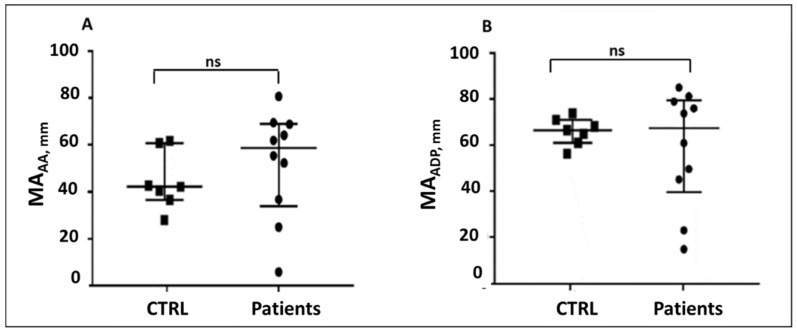
(**A**,**B**) Comparison of median and interquartile range of MA_AA_ and MA_ADP_ between controls and patients at T0.

In contrast, measurement of the maximal amplitude (MA) parameter revealed a heterogeneous distribution of MA values among patients compared to controls at T0. Some patients displayed higher MA_ADP_ and MA_AA_ values, and others displayed lower values compared to controls. These findings suggest that, at the time of enrollment, the MA parameter identified both patients with increased and patients with impaired clot firmness, as this parameter was most probably influenced by other factors such as fibrinogen and inflammatory mediators in sepsis/septic shock patients (Figure 4A,B).

**Figure 5:**
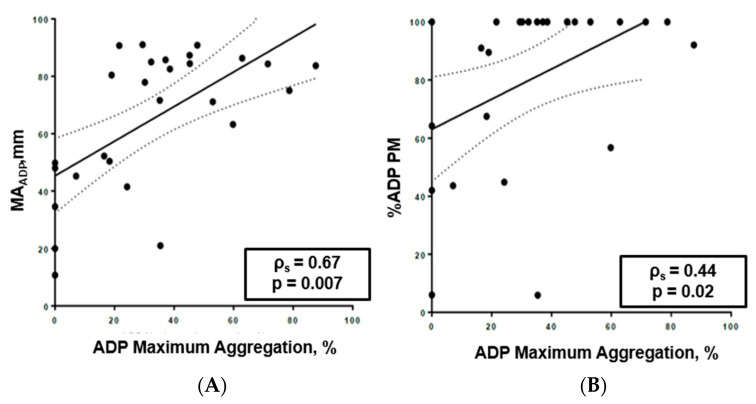
(**A**,**B**): (**A**) Correlation between MA_ADP_ and ADP aggregation measured by LTA at three time points. (**B**) Correlation between platelet aggregation rate at Platelet Mapping (%ADP PM) and ADP aggregation rate measured by LTA (ADP maximum aggregation, %) at three time points.

The results obtained from the viscoelastic tests with ADP (MAADP and platelet ag-gregation rate—%ADP PM) were compared with the ADP aggregation rate measured by LTA (ADP maximum aggregation) at the same time, showing a correlation between the two datasets, which was stronger and more evident with MAADP (Figure 5A,B).

### 3.5. Flow Cytometry Measurements of Pac1 and P-Selectin Expression

**Figure 6:**
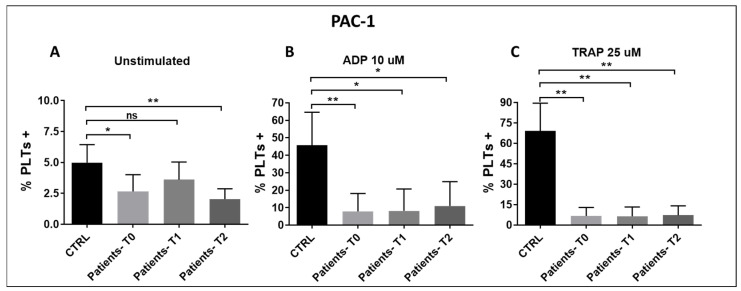
(**A**–**C**): PAC1 binding to unstimulated (**A**), ADP-stimulated (**B**), and TRAP-stimulated (**C**) platelets of patients with sepsis and septic shock compared to healthy controls at T0, T1, and T2. Results are expressed as % of PAC-1 positive platelets, identified as CD42B-positive events. (**A**) T0 vs. CTRL: p*_bonf_* = 0.021; T1 vs. CTRL: p*_bonf_* = 0.12; T2 vs. CTRL: p*_bonf_* = 0.0009; (**B**) T0 vs. CTRL: p*_bonf_* = 0.0012; T1 vs. CTRL: p*_bonf_* = 0.0063; T2 vs. CTRL: p*_bonf_* = 0.0063 (**C**) T0 vs. CTRL: p*_bonf_* = 0.0003; T1 vs. CTRL: p*_bonf_* = 0.0006; T2 vs. CTRL: p*_bonf_* = 0.0006. Bonferroni correction was applied for all pairwise compari-sons; *p*-values (p*_bonf_*) are reported. Sample size: n = 10 at T0 and T2, n = 9 at T1. * *p* < 0.05, ** *p* < 0.005. ns = no significance.

**Figure 7:**
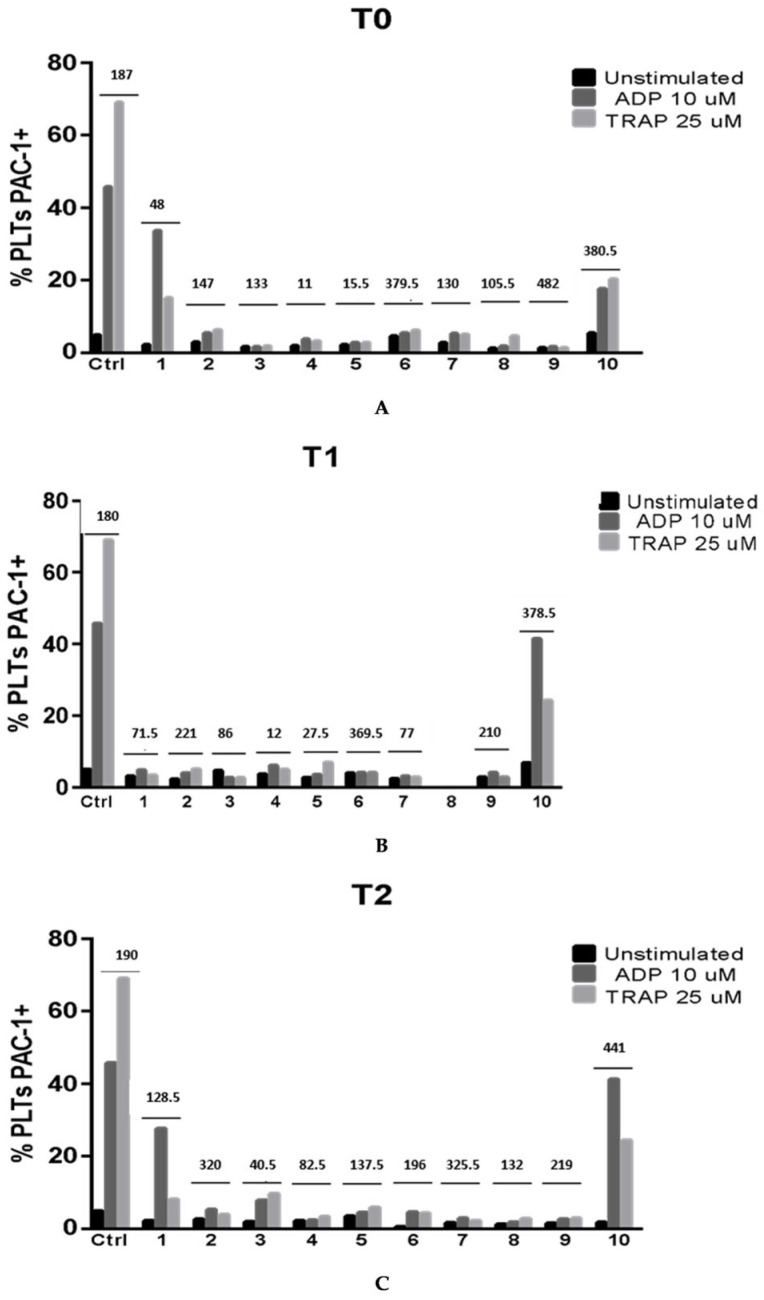
(**A**–**C**) Individual expression of fibrinogen receptor, as measured by PAC-1 binding, on unstimulated, ADP-stimulated, and TRAP-stimulated platelets of the consecutively enrolled sepsis and septic shock patients #1–10 at T0, T1, and T2 compared to controls. T1 was not performed in patient #8. The numbers above the graphs indicate whole platelet count measured at each time (× 103/µL).

PAC-1 is a monoclonal antibody that specifically recognizes the activated confor-mation of the fibrinogen receptor GPIIb/IIIa (also called integrin αIIbβ3). Hence, meas-uring PAC-1 binding by flow cytometry is a way to assess platelet activation capacity at the receptor level. PAC-1 binding was severely reduced in ADP- and TRAP-stimulated platelets of sepsis/septic shock patients (Figure 6A–C). Notably, this pattern was measured in all 10 patients at all time points (Figure 7). PAC-1 expression was severely affected at T0 already, and it was independent of thrombocytopenia, as it was also documented in the presence of high platelet count, as shown for patients #6, #9, and #10 (Figure 7).

**Figure 8:**
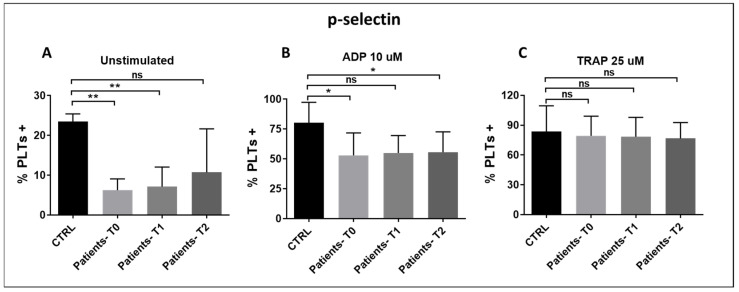
(**A**–**C**): P-selectin expression on unstimulated (**A**), ADP-stimulated (**B**), and TRAP-stimulated (**C**) platelets of patients with sepsis and septic shock compared to healthy controls at T0, T1, and T2. Results are expressed as a % of P-selectin-positive platelets, identified as CD42 B-positive events. (**A**) T0 vs. CTRL: p*_bonf_* = 0.0006; T1 vs. CTRL: p*_bonf_* = 0.0006; T2 vs. CTRL: p*_bonf_* = 0.12; (**B**) T0 vs. CTRL: p*_bonf_* = 0.026; T1 vs. CTRL: p*_bonf_* = 0.056; T2 vs. CTRL: p*_bonf_* = 0.037; (**C**) n.s.= not significant. Bonferroni correction was applied for all pairwise comparisons; *p*-values (p*_bonf_*) are reported. Sample size: n = 10 at T0 and T2, n = 9 at T1. * *p* < 0.05, ** *p* < 0.005. ns = no significance.

P-selectin expression on the membrane of activated platelets is a marker of platelet activation and reflects α-granule secretion. The expression of P-selectin on the platelet surface was significantly reduced in patients in response to ADP and on unstimulated platelets (Figure 8A,B). On the contrary, P-selectin expression after TRAP stimulation was not significantly different in patients compared to controls (Figure 8C). A possible explanation is that, in the context of sepsis or septic shock, P-selectin may be a less sensitive marker of platelet reactivity than PAC1 expression. Alternatively, the TRAP concentration used in our internal flow cytometry protocol may have been too high to detect potential differences between patients and controls.

### 3.6. Plasma Levels of Soluble P-Selectin and Cd40l

**Figure 9:**
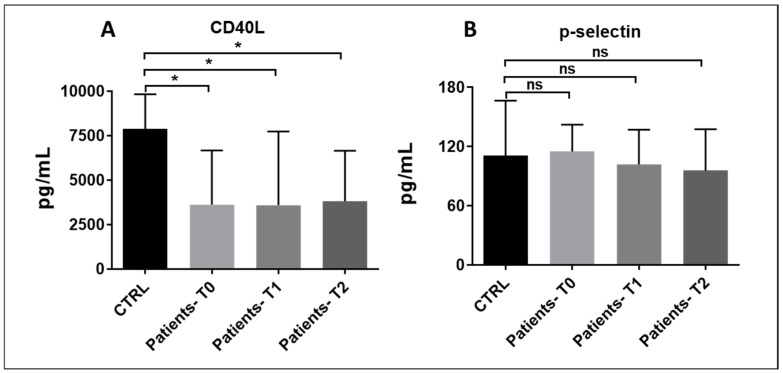
(**A**,**B**): Patient soluble CD40L (**A**) and soluble P-selectin (**B**) plasma levels at times T0, T1, and T2 compared to controls. (**A**) T0 vs. CTRL: p*_bonf_* = 0.05; T1 vs. CTRL: p*_bonf_* = 0.04; T2 vs. CTRL: p*_bonf_* = 0.018. Bonferroni correction was applied for all pairwise comparisons; *p*-values (p*_bonf_*) are re-ported. Sample size: n = 10 at T0 and T2, n = 9 at T1. * *p* < 0.05. ns = no significance.

Plasma levels of CD40 ligand in septic patients were significantly lower than those of controls at all time points (Figure 9A). On the contrary, soluble plasma levels of P-selectin showed no differences between the two groups (Figure 9B).

### 3.7. Platelet–Leukocyte Aggregates

**Figure 10:**
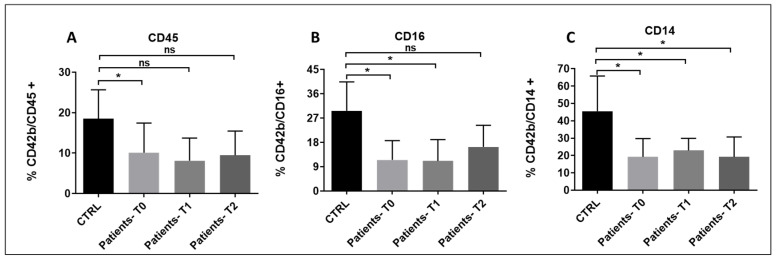
(**A**–**C**): Platelet–leukocyte aggregates in sepsis and septic shock patients at times T0, T1, and T2 compared to controls. (**A**) T0 vs. CTRL p*_bonf_* = 0.033; T1 vs. CTRL: p*_bonf_* = 0.06; T2 vs. CTRL: p*_bonf_* = 0.075; (**B**) T0 vs. CTRL: p*_bonf_* = 0.0036; T1 vs. CTRL: p*_bonf_* = 0.0066; T2 vs. CTRL: p*_bonf_* = 0.069; (**C**): T0 vs. CTRL: p*_bonf_* = 0.0087; T1 vs. CTRL: p*_bonf_* = 0.027; T2 vs. CTRL: p*_bonf_* = 0.034. Sample size: n = 10 at T0 and T2, n = 9 at T1. Bonferroni correction was applied for all pairwise comparisons; *p*-values (p*_bonf_*) are reported. * *p* < 0.05. ns = no significance.

The number of all three types of platelet–leukocyte aggregates was significantly lower in patients compared to controls at T0. Platelet/monocyte aggregates were significantly reduced at all three time points, and a trend toward reduction was measured for platelet/neutrophil aggregates as well (Figure 10). These results might reflect immune and platelet exhaustion already present at the onset of the disease. In this state, both leukocytes and platelets become dysfunctional and are less likely to form aggregates. Another plausible explanation—aligned with elevated von Willebrand factor (vWF) levels—is that endothelial damage in sepsis promotes platelet and leukocyte adhesion to the vessel wall, thereby reducing the formation of circulating platelet–leukocyte aggregates.

### 3.8. Von Willebrand Factor Plasma Levels

**Figure 11:**
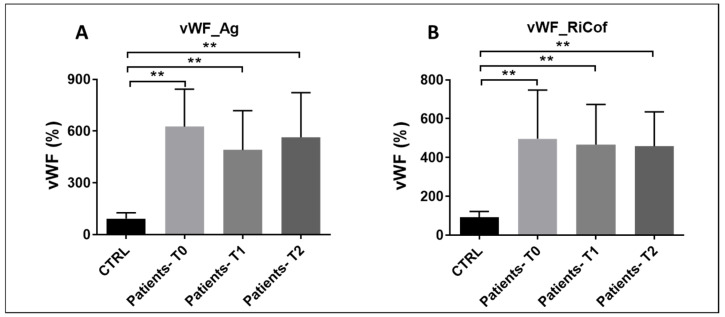
(**A**,**B**): vWF_Ag (**A**) and vWF ristocetin cofactor (RiCof) (**B**) plasma levels in patients with sepsis and septic shock compared to controls at T0, T1, and T2. (**A**): T0 vs. CTRL: p*_bonf_* = 0.0003; T1 vs. CTRL: p*_bonf_* = 0.0006; T2 vs. CTRL: p*_bonf_* = 0.0003; (**B**): T0 vs. CTRL: p*_bonf_* = 0.0036; T1 vs. CTRL: p*_bonf_* = 0.0006; T2 vs. CTRL: p*_bonf_* = 0.0003. Sample size: n = 10 at T0 and T2, n = 9 at T1. Bonferroni correc-tion was applied for all pairwise comparisons; *p*-values (p*_bonf_*) are reported. ** *p* < 0.005.

Plasma levels of von Willebrand factor antigen and ristocetin cofactor were much higher (5- to 6-fold) in septic patients at all time points compared to controls, with a slight, not significant trend toward reduction at T1 and T2 (Figure 11A,B). von Willebrand factor, one of the main indicators of vascular endothelial cell injury and dysfunction, plays a crucial role in the pathophysiology of sepsis.

### 3.9. Correlation of Platelet Results with Sepsis Markers and Clinical Sepsis Param-Eters

Viscoelastic and platelet aggregation test parameters, as well as fibrinogen receptor expression via PAC-1 (with and without stimulation), were correlated with SOFA score and sepsis markers (procalcitonin) for each patient at each study time point. No correlation was found between SOFA score, procalcitonin, and fibrinogen receptor expression in stimulated platelets. On the contrary, the expression of the fibrinogen receptor measured by PAC-1 binding in unstimulated platelets showed the following correlations, displayed in Table 2.

**Figure 12:**
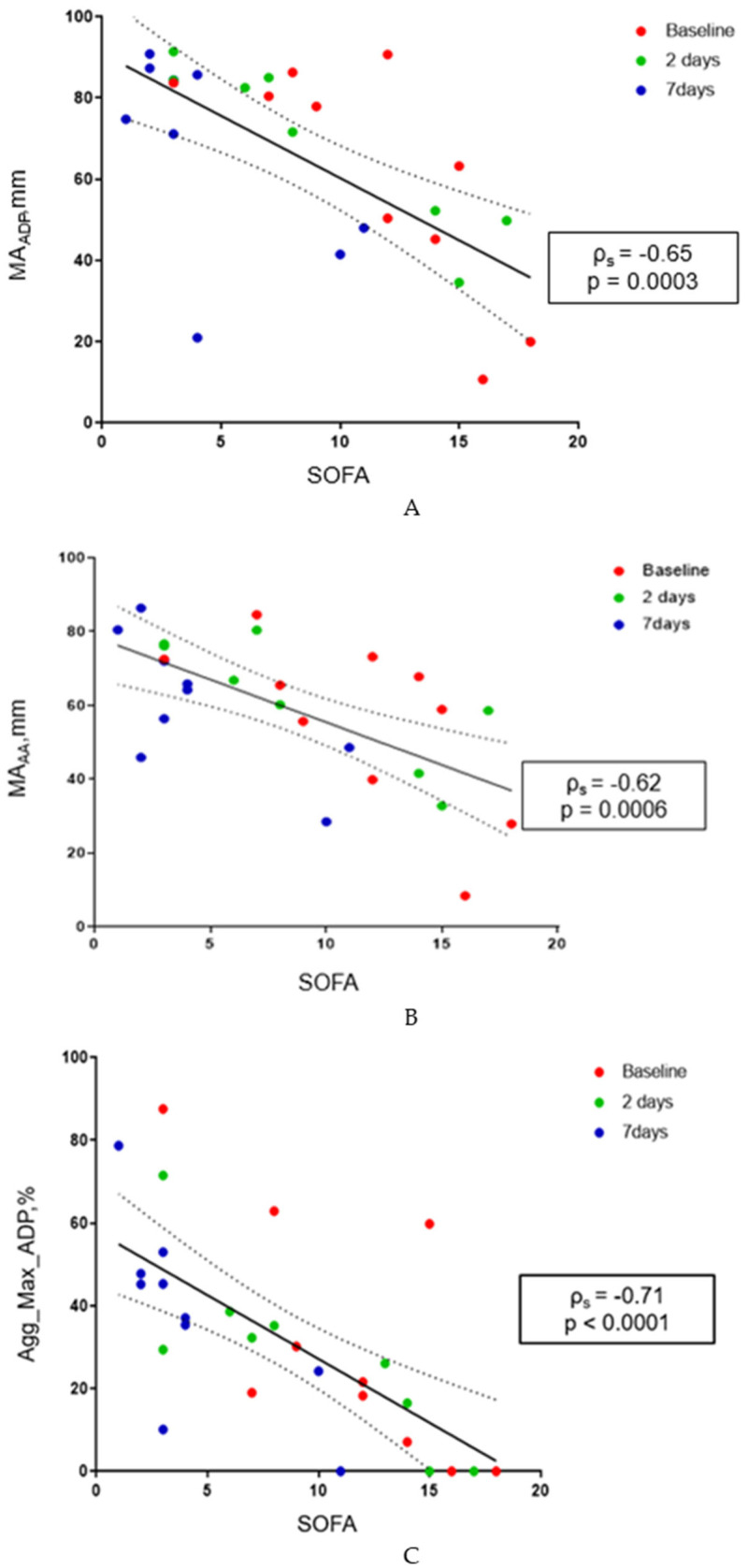
(**A**–**C**): (**A**) Correlation between MA_ADP_ and SOFA score at three time points. (**B**) Correlation between MA_AA_ and SOFA score at three time points. (**C**) Correlation between ADP-induced maximal aggregation as measured by LTA (Agg_max_ADP%) and SOFA score at three time points.

**Figure 13:**
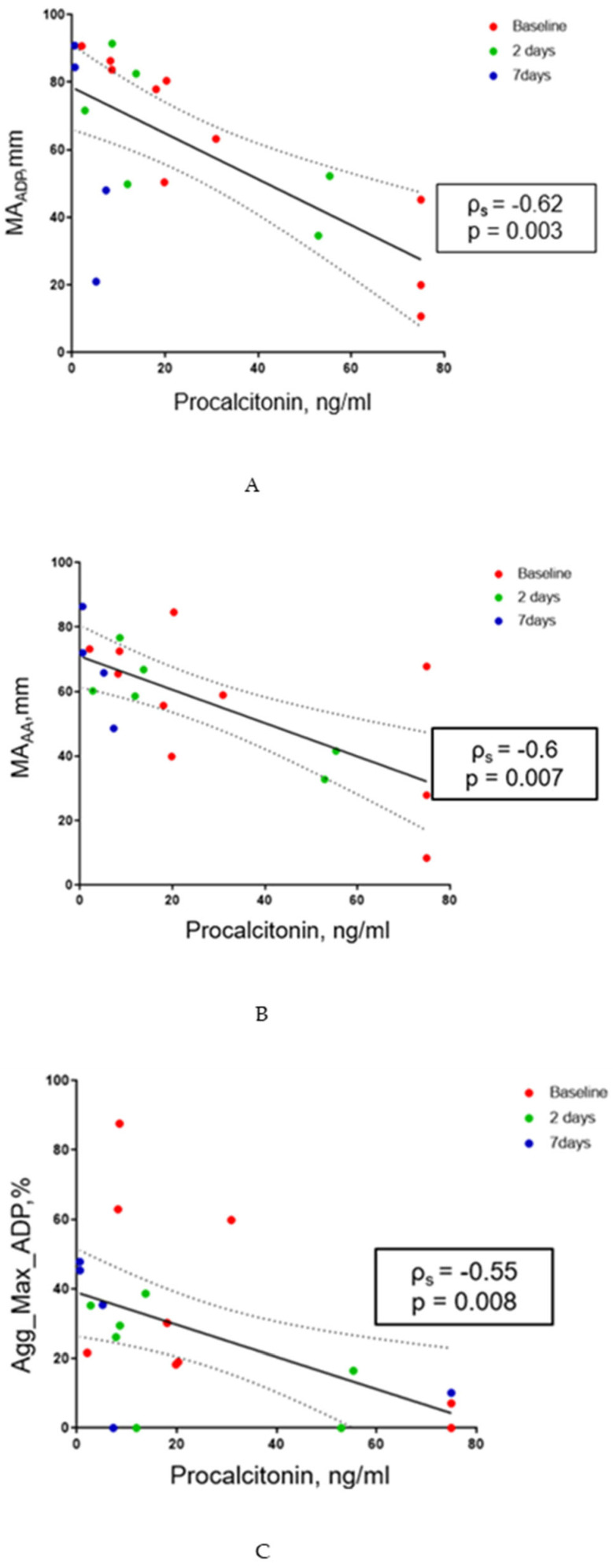
(**A**–**C**): (**A**) Correlation between MA_ADP_ and procalcitonin levels at three time points. (**B**) Correlation between MA_AA_ and procalcitonin levels at three time points. (**C**) Correlation between ADP-induced maximal aggregation as measured by LTA (Agg_max_ADP%) and procalcitonin levels at three time points.

The strongest correlations were observed across T0, T1, and T2 between SOFA and the viscoelastic parameters MA_ADP_ (ρ_s_ = −0.65, *p* = 0.0003) and MA_AA_ (ρ_s_ = −0.62, *p* = 0.0006), and between SOFA and ADP-induced aggregation measured by LTA (ρ_s_ = −0.71, *p* < 0.0001). Also, strong correlations were measured between procalcitonin and the viscoelastic parameters MA_ADP_ (ρ_s_ = −0.62, *p* = 0.003) and MA_AA_ (ρ_s_ = −0.6, *p* = 0.007), as well as between procalcitonin and ADP-induced aggregation measured by LTA (ρ_s_ = −0.55, *p* = 0.008) (Figure 12 and Figure 13). Correlations at T0 are summarized in Table 3.

## 4. Discussion

This pilot study provides novel insights into the early hemostatic alterations occurring in patients with sepsis and septic shock. Specifically, we demonstrated that significant platelet dysfunction can be detected even when platelet counts remain within the normal range, challenging the conventional reliance on platelet count as a standalone marker of hemostatic status in clinical practice. We also demonstrated impairment of certain pathways involved in platelet responses to pathogen infections. Our findings suggest that functional platelet impairment emerges early in the course of the disease and may go undetected without the use of dedicated functional assays.

Most studies on sepsis have focused on thrombocytopenia, which is recognized as an early marker of sepsis and is associated with poor outcomes. In the present study, not only did we not observe severe thrombocytopenia in a time range of 7 days from diagnosis, but we also recorded the lowest platelet count only 48 h after admission to the ICU.

In contrast, we observed a significant reduction in platelet function in sepsis and septic shock patients, regardless of platelet count and despite the limitations of a small sample size. Platelet hemostatic hyporeactivity was observed using multiple comple-mentary approaches. LTA, viscoelastic tests measuring ADP- and AA-induced platelet aggregation, and assessment of activated fibrinogen receptor exposure by PAC-1 binding all demonstrated a marked reduction in hemostatic platelet function among sepsis and septic shock patients. The severe defect in platelet function was independ-ent of thrombocytopenia and was observed even in patients with normal or elevated platelet counts. Moreover, it could be detected very early, being already present at the time of ICU admission.

LTA performed with ADP and epinephrine stimulation showed significantly re-duced platelet aggregation at admission, which persisted for at least seven days, alt-hough a trend toward improvement was documented by day seven. These results were in agreement with previously reported defects of platelet aggregation [33,34]. It has to be highlighted that we performed platelet aggregation only when platelet count was >150 × 10^9^/L, according to ISTH [35], thus ruling out any spurious effect of thrombocy-topenia on LTA values. Platelet function assessed by Platelet Mapping also demon-strated a significant reduction in aggregation capacity, as indicated by the percentage of platelet aggregation in response to both ADP and AA stimulation. Interestingly, the correlation between the platelet function measured by viscoelastic tests and by LTA highlights the role of viscoelastic tests for fast investigation of platelet function in the context of emergency departments. Hence, the present study supports the use of the viscoelastic test as a valuable tool for assessing platelet function in patients with sepsis and septic shock.

Since thrombocytopenia represents a major methodological challenge in assessing platelet function, it significantly limits the opportunity to investigate platelet dysfunc-tion in a large proportion of patients with sepsis and septic shock. We employed a platelet count-independent method, i.e., flow cytometry, as previously described by other authors [33].

The most severe platelet defect observed in patients with sepsis and septic shock was a marked reduction in fibrinogen receptor expression, as measured by PAC-1 binding following stimulation with both ADP and TRAP. In these patients, fibrinogen receptor expression was significantly decreased not only in resting platelets but also after in vitro stimulation. Notably, this severe impairment in PAC-1 binding was consistently observed across all patients and persisted up to seven days after hospital admission. This sustained alteration may represent a potential early biomarker of sepsis and a risk factor for adverse clinical outcomes. Further investigation is warranted to determine whether the persistence of this abnormality correlates with disease severity or prognosis. Our results are consistent with previous studies that assessed fibrinogen receptor activation—measured by PAC-1 or fibrinogen binding to platelets using flow cytometry—in sepsis patients compared to healthy controls [11,12,33]. Notably, we showed that these functional impairments were observed even in patients with normal or elevated platelet counts, highlighting that quantitative platelet levels may not reflect their functional status in sepsis.

We also found a reduced percentage of P-selectin-positive platelets in both quies-cent and ADP-stimulated platelet conditions, in agreement with the study by Laursen [33], but not in platelets stimulated with 25 uM TRAP. This latter result corroborates the study by Yaguchi et al. [11], who reported no difference in P-selectin expression between septic patients and controls after platelet stimulation with TRAP 50 uM and collagen 2.5 ug/mL [36,37]. The lack of significant difference in P-selectin expression in TRAP-stimulated platelets might depend on high concentrations of the agonist used in both our study and that of Yaguchi et al., which could have been insufficient to mask any differences between the two groups.

The above-reported results confirm previous findings of platelet hemostatic hyporeactivity in sepsis patients. The most likely explanation for the above findings is that prolonged or repetitive platelet activation can result in functional exhaustion, characterized by reduced responsiveness to subsequent agonist stimulation despite preserved platelet counts. This state may arise from direct activation by pathogen-associated molecular patterns (PAMPs), such as bacterial lipopolysaccharide, that can directly engage platelet Toll-like receptors, triggering repeated activation, receptor desensitization, altered signaling, and surface receptor shedding and degranulation, as well as from indirect activation via inflammatory cytokines and chemokines—particularly IL-6, TNF-α, and CXCL4—which induce chronic platelet priming followed by signaling dysregulation and impaired secretion, thus further amplifying platelet stimulation [38,39]. Repeated activation also promotes granule depletion and metabolic stress, further contributing to diminished platelet aggregation and function [15,40]. These processes result in reduced platelet responsiveness to physiological agonists, reflecting a state of “platelet exhaustion” that may contribute to the bleeding tendency and impaired hemostatic balance observed in severe sepsis.

Surprisingly, soluble plasma P-selectin levels, reflecting platelet activation and alpha-granule release in plasma, were not different in sepsis patients compared to controls. We expected higher soluble P-selectin levels as a consequence of platelet ac-tivation in vivo and exhaustion. Laursen et al. [33], in fact, reported increased levels of soluble P-selectin in septic shock patients compared to controls during the first three days from the onset of the disease. Our findings might reflect platelet hyporeactivity that was already present at admission, possibly resulting from prior platelet exhaus-tion induced by the infectious process.

Platelet function abnormalities have important clinical implications. Interestingly, we observed a correlation between hemostatic platelet dysfunction and markers of sepsis severity. Specifically, the degree of platelet impairment, measured with MA_ADP_ and MA_AA_, was negatively correlated with both SOFA score and procalcitonin levels. Likewise, ADP aggregometry showed a correlation with SOFA score and with procalcitonin. Higher SOFA scores and procalcitonin levels were associated with reduced platelet aggregation as assessed by Platelet Mapping and ADP LTA, particularly at time point T0.

We also demonstrated impairment of some pathways involved in platelet responses to pathogen infections.

For instance, membrane-bound P-selectin has been extensively studied not only as a key marker of platelet activation but also for its critical role in the formation of plate-let–leukocyte aggregates, which play an essential part in immunothrombosis [21]. The binding of P-selectin to PSGL-1 is essential for the formation of aggregates between platelets and neutrophils, as well as for neutrophil extravasation [14,41]. CD40L is also a platelet-derived molecule and was the first to reveal the role of platelets as immune cells [42]. Platelets are the primary source of soluble CD40L, which binds to CD40 re-ceptors on leukocytes and initiates immune responses. CD40L stabilizes platelet–leukocyte aggregates and amplifies inflammatory signaling, thereby promoting im-mune defense and contributing to immunothrombosis. Liu et al. reported increased levels of soluble CD40L in sepsis patients [43], whereas other studies have shown a significant reduction [44]. In the present study, we found a significant reduction in both membrane-bound P-selectin expression on platelets from sepsis and septic shock patients and plasma soluble CD40L levels, together with a significant decrease in cir-culating platelet–neutrophil aggregates. These changes may reflect platelet exhaustion, which may ultimately result in impaired platelet activation and immune function in patients with sepsis and septic shock. Although we cannot provide a definitive expla-nation for these findings, it is plausible that the inhibition of the host’s innate immune response is driven by mechanisms initiated by the invading microorganisms. These may involve pathogen-associated molecular patterns (PAMPs) or damage-associated molecular patterns (DAMPs), which modulate host immunity and may contribute to the pathogenesis of sepsis.

Taken together, our findings support the existence of a functional link between impaired platelet activation and early immune dysregulation in sepsis, suggesting that the weakening of host immune defense begins at an early stage of the disease.

Another important phenomenon in sepsis is endothelial activation. We measured plasma levels of von Willebrand factor (vWF) and found them to be markedly elevated in patients with sepsis or septic shock, reflecting massive endothelial activation. Notably, these levels remained persistently elevated up to seven days following hospital admission. This observation is consistent with previously reported data [44].

A novel aspect of this study that we would like to emphasize is the comprehensive evaluation of platelet dysfunction from complementary perspectives, using both a viscoelastic method—such as Platelet Mapping—and the reference standard, light transmission aggregometry (LTA). To our knowledge, this is the first study that investigates the correlation between Platelet Mapping and LTA in assessing platelet function in the context of sepsis and septic shock. This finding is particularly relevant for intensive care settings, where rapid, bedside-compatible methods such as Platelet Mapping could substantially reduce diagnostic time compared with more labor-intensive techniques like LTA. In the ICU setting, viscoelastic tests not only provide the opportunity for rapid assessment of platelet function but also offer a comprehensive evaluation of the entire coagulation process, delivering more precise insights into hemostatic changes. Sepsis and septic shock patients exhibit a range of viscoelastic profiles, which may vary based on disease severity and sampling timing. Conflicting results have been reported regarding the hemostatic pattern in sepsis and septic shock, as well as the underlying pathophysiological mechanisms. Studies with viscoelastic tests (TEG/ROTEM) report variable results with both hypo-coagulative and hyper-coagulative patterns described [45,46,47,48,49]. Indeed, using the Platelet Mapping assay on TEG, we also show a heterogeneous distribution of the MA parameter, which reflects overall clot strength depending not only on platelet aggregation but also on platelet–fibrin interaction, fibrinogen levels, and fibrin polymerization. Hence, this parameter could identify patients with increased and patients with impaired clot firmness with respect to controls in our cohort.

An interesting finding is the linear association between impaired hemostasis and the severity of organ damage [46]. In a study comparing hemostatic competency assessed by TEG across different patient populations, e.g., surgery, trauma, and sepsis patients, impairment was particularly evident in patients with sepsis, where reduced hemostatic competency was associated with more severe organ failure [48]. As supported by the evidence in the literature, changes in platelet function, either hyper-reactive or hypo-reactive, are linked to increased mortality in the context of sepsis and septic shock. In particular, mortality rises by approximately 40% with reduced platelet function and by 10% with increased function [34,45,50,51]. Our study was not designed to correlate laboratory data with clinical outcome; thus, we could not provide data on the mortality.

It should be emphasized that the timing of viscoelastic test measurements is crucial, as the hypocoagulative pattern typically appears in the early stages of sepsis, as also observed in our study, and tends to normalize by the time of ICU discharge [46,49]. Another explanation for the variability in viscoelastic profiles in sepsis and septic shock patients lies in the lack of standardized criteria for result interpretation, as well as the absence of clear definitions for hypocoagulable and hypercoagulable states. To date, only one study has established cut-off values for sepsis patients [51]. Others have classified results based on deviations from manufacturer-provided reference ranges [45,52,53] or by comparison with healthy volunteer data [47,54,55,56]. These two typical patterns still require further investigation, and additional studies are needed to better define clinically relevant cut-off values.

The clinical implications of our findings are notable. First, the presence of severe hemostatic platelet dysfunction despite normal or even elevated platelet counts may help identify patients at higher risk of bleeding, which could otherwise go unrecognized. Second, the marked reduction in platelet/leukocyte aggregates observed in our septic cohort may indicate a state of immune dysregulation. Given the known role of platelet–leukocyte interactions in modulating immune responses, this reduction could be associated with a worse prognosis, potentially due to impaired host defense mechanisms and increased susceptibility to secondary infections.

Taken together, our findings highlight the potential utility of early functional assessment of platelet activity in septic patients. This approach may provide diagnostic and prognostic value beyond that offered by standard coagulation parameters and platelet counts, and it sets the stage for larger studies aimed at validating these preliminary observations in broader patient populations.

## 5. Limitations

The main limitation of our study is the small sample size and the lack of longitudinal stratification between sepsis and septic shock subgroups. First, enrollment of participants was difficult due to the common use of antiplatelet agents. Second, the highly specialized and resource-intensive nature of the multiple platelet function assays we employed limited the number of patients we could include in this initial exploratory study. We acknowledge that the limited number of patients prevents us from drawing definitive conclusions. However, our study also has some strengths. Platelet dysfunction was consistently demonstrated through multiple, independent methodological approaches. For the first time, viscoelastic tests and the gold-standard test for platelet function studies were compared. The study assessed both platelet hemostatic and immune functions. Despite the exploratory nature of our results, we believe they provide a compelling rationale to further investigate early platelet dysfunction in larger, prospective studies.

## 6. Conclusions

Our findings demonstrate a significant reduction in hemostatic platelet function as measured by LTA, viscoelastic methods, and agonist-induced P-selectin and fibrinogen receptor exposure in patients with sepsis and septic shock. This platelet hyporeactivity persisted for up to 7 days from diagnosis and was found to be independent of platelet count. The use of bedside viscoelastic tests provides a valuable complement to gold-standard assays of platelet function, such as LTA. Platelet dysfunction is closely linked to the severity of sepsis, particularly during its onset, suggesting that it may serve as an early marker of the disease, potentially more reliable than platelet count alone. Moreover, platelet hyporeactivity might reflect an increased bleeding risk in these patients, independent of thrombocytopenia. We also demonstrated impairment of certain platelet-dependent immune responses to pathogens. Reduced platelet–leukocyte aggregates in sepsis and septic shock patients may reflect exhaustion of platelet function and of leukocyte responses to microorganisms, potentially contrib-uting to impaired host defense during infection. Future studies are warranted to ex-plore whether targeting the mechanisms causing platelet dysfunction could improve clinical outcomes.

## Figures and Tables

**Table 1:** Baseline characteristics of enrolled patients and controls at T0. Continuous variables are expressed as median and interquartile range. Categorical variables are expressed as percentages.

Baseline Characteristics of Enrolled Patients and Controls at T0	
No. of patients/controls	10/7
Age, years	61 (49–72)/31 (30.5–33.5)
Female sex, no. (%)	5 (50%)/3 (43%)
Height, cm	165 (163–175)/175 (168–187)
Weight, kg	85 (67–110)/76 (61–84)
BMI, kg/m^2^	28.08 (22.91–39.18)/23,77 (21.48–24.59)
SAPS II—T0	56 (50–80)
SOFA—T0	12 (8–15)
White Blood Cell/µL	18,280 (6990–32,830)
Hematocrit, %	31.9 (26.9–33.7)
Platelet count, 10^3^/µL	227 (99–459)
Serum creatinine, mg/dL	2.66 (1.62–4.16)
Blood urea nitrogen, mg/dL	31 (21–53)
Total serum bilirubin, mg/dL	2.20 (0.7–4.20)
INR	1.51 (1.22–2.59)
Plasma fibrinogen, mg/dL	564.5 (336.25–764)
Serum lactates, mmol/L	5.9 (4.1–7.1)
HCO_3_^−^, mmol/L	15.3 (12.6–19)
PH	7.37 (7.26–7.43)
Serum procalcitonin, ng/mL	19.83 (8.25–75)
Serum C-reactive protein, mg/dL	175 (154.45–280.9)
Mean arterial pressure, mmHg	54 (52–65)

**Table 2:** Correlation between expression of the fibrinogen receptor measured by PAC-1 binding in unstimulated platelets and procalcitonin at T1 and T2.

T1	T2
Procalcitonin, ng/mL ρ_s_ = 0.82, *p* = 0.03	Procalcitonin, ng/mL ρ_s_ = 0.97, *p* = 0.03

**Table 3:** Correlation between SOFA score, procalcitonin levels, viscoelastic tests, and LTA at T0 in sepsis and septic shock patients.

T0	SOFA	Procalcitonin, ng/mL
MA_ADP_	ρ_s_ = −0.76 *p* = 0.014	ρ_s_ = −0.84 *p* = 0.02
MA_AA_	ρ_s_ = −0.69 *p* = 0.032	ρ_s_ = −0.51 *p* = 0.17
ADP Maximum Aggregation [%]	ρ_s_ = −0.72 *p* = 0.023	ρ_s_ = −0.74 *p* = 0.01

## Data Availability

All available data are shown in the manuscript.

## Abbreviations

The following abbreviations are used in this manuscript:

LTA	Light Transmission Aggregometry
TEG	Thromboelastography
MOF	Multi-Organ Failure
DIC	Disseminated Intravascular Coagulation
MODS	Multi-Organ Dysfunction Syndrome
SAPS 2	Simplified Acute Physiology Score 2
SOFA	Sepsis-related Organ Failure Assessment
MA	Maximum Amplitude
AA	Arachidonic Acid
PM	Platelet Mapping
BMI	Body Mass Index
CI	Coagulation Index
Agg_max_ADP	ADP Maximum Aggregation by LTA
